# Arylamine N-acetyltransferase 2 Polymorphisms and the Risk of Endometriosis

**Published:** 2018

**Authors:** Diman Fayez, Kioomars Saliminejad, Shiva Irani, Koorosh Kamali, Toktam Memariani, Hamid Reza Khorram Khorshid

**Affiliations:** 1. Department of Biology, Faculty of Science, Science and Research Branch, Islamic Azad University, Tehran, Iran; 2. Reproductive Biotechnology Research Center, Avicenna Research Institute, ACECR, Tehran, Iran; 3. Central Research Lab, North Khorasan University of Medical Sciences, Bojnurd, Iran; 4. Genetic Research Center, University of Social Welfare and Rehabilitation Sciences, Tehran, Iran

**Keywords:** Endometriosis, Genome wide association study, *NAT2*, Polymorphism

## Abstract

**Background:**

Human arylamine N-acetyltransferase 2 *(NAT2)* gene has a key role in xenobiotic metabolism through the conjugation of acetyl group to xenobiotic substances. *NAT2* has been suggested as a susceptibility factor in endometriosis; however, the results of studies have been controversial. In this study, the association of *NAT2* polymorphisms with susceptibility to endometriosis was evaluated in an Iranian population.

**Methods:**

This is an association study and totally 141 women with diagnosis of endometriosis and 158 healthy women as control group were analyzed for *NAT2* gene polymorphisms (C481T, A803G, G857A and G590A) by PCR-RFLP methods.

**Results:**

The 590 GA genotype was significantly lower (p=0.001; OR=0.42, 95% CI: 0.25–0.71) in the patients (38.3%) than the control group (55.1%). The 590A allele was significantly lower (p=0.033; OR=0.69, 95% CI: 0.49–0.79) in the patients (31.2%) compared with the controls (39.6%). Analysis of haplotypes showed that *NAT2* 481C, 803A, 590A, 587A combination was significantly different between the case and control women (p= 0.029; OR=3.11, 95% CI: 1.13–8.52).

**Conclusion:**

The *NAT2* G590A SNP may be associated with susceptibility to endometriosis and the 590A allele may have a protective role in development of endometriosis. The *NAT2* 481C, 803A, 590A, 587A haplotype was associated with a higher risk of endometriosis in Iranian population.

## Introduction

Endometriosis, a benign gynecological and estrogen dependent disease, is the growth of endometrium outside the uterine cavity or myometrium, usually in the peritoneal cavity 1–3. The most important symptoms of endometriosis are back pain, infertility, dyspareunia, dysmenorrheal, dyschezia and pelvic pain 4. Several factors such as genetic, immune, endocrine and environmental factors have been associated with the pathogenesis of endometriosis 1,5–10.

The human arylamine N-acetyltransferase 2 *(NAT2)* gene, on chromosomal region 8p22 11, has a key role in conjugation of xenobiotic substances. *NAT2* is involved in the initial biotransformation metabolism of aromatic amines and hydrazines, and catalyzes the transfer of an acetyl group from acetyl CoA to the nitrogen of the substrate 12. Some polymorphisms in *NAT2* lead to amino acid substitution, which may result in impairment of the enzyme activity. Regarding *NAT2*, individuals are classified as rapid metabolizers if they have one or two wild-type alleles such as *NAT2* *4 which is the most common allele. On the other hand, individuals are classified as slow metabolizers only if they carry two slow metabolizer variants such as *NAT2* *5,*6 or *7 alleles. In a paper by Hein *et al*, they determined the NAT gene nomenclature 13. The alleles themselves are effectively haplotypes composed of several *NAT2* polymorphisms, most typically assigned according to the status of the seven polymorphisms. *NAT2* slow acetylation has been reported as a risk factor for bladder cancer 14,15, while fast acetylation has been implicated as a risk factor for colon cancer 16. Smelt and Mardon showed that there was a linkage disequilibrium between NAT1 and *NAT2* genes 17.

The results of previous studies that evaluated the *NAT2* polymorphisms with the risk of endometriosis are controversial. Therefore, in the current study, it was decided to investigate the association of *NAT2* C481T (rs1799929), G590A (rs1799930), A803G (rs1208) and G857A (rs1799931) polymorphisms with endometriosis in an Iranian population.

## Materials and Methods

### Subjects

In this case-control study, 141 Iranian patients with diagnosis of endometriosis were included. The diagnosis of endometriosis was accurately predicted preoperatively by clinicians. The severity of the disease (Stage I–IV) was scored according to the revised American Society for Reproductive Medicine (ASRM) classification. As the control group, 158 age-matched healthy women without endometriosis were selected. Absence of the endometriosis in the control group was confirmed by laparoscopic abdominal examination to inspect conditions other than endometriosis. Cases without confirmation of the disease by laparoscopy were excluded. Approval from Avicenna Research Institute’s Ethics Committee was obtained. Written informed consent was received from the patients and controls. The study was done at Avicenna Research Institute, Tehran, Iran.

### Genotype and phenotype determination

DNA was extracted from peripheral blood samples according to the salting out procedure. Genotyping of the four polymorphisms were performed using PCR-RFLP methods according to the previous study 16. Briefly, 546 *bp* fragment from exon 2 of *NAT2* was amplified using the forward primer 5′-GCTGGTCTGG AAGCTCCTC-3′ and reverse primer 5′-TTGGGTGA TACATACACAAGGG-3′. Following PCR amplification, digestion with the restriction enzymes KpnI, Dd-eI, BamHI and TaqI were performed to detect genotypes of the 481C/T, 803A/G, 857G/A and 590G/A polymorphisms, respectively. All enzymes were obtained from New England BioLabs and were used according to the manufacturer’s instructions.

*NAT2* acetylator phenotypes (slow, intermediate and fast) were predicted using the NAT2PRED Web server available at http://nat2pred.rit.albany.edu 18. NAT2P-RED is a web-server that predicts *NAT2* acetylator phenotype from six polymorphisms of *NAT2* C282T, T341C, C481T, A803G, G857A and G590A, without taking the extra step of reconstructing haplotypes for each individual 19. Because the genotypes for the *NAT2* C282T and T341C were not determined, the wild type genotype for these polymorphisms was selected in the NAT2PRED web server.

### Statistical analysis

Statistical analysis was performed with SPSS for Windows, version 13 (Chicago, IL, USA). Analyses of the allele frequencies and genotype distributions for the *NAT2* polymorphisms in case and control groups were performed by *χ*^2^ test and logistic regression analysis. All p-values were reported following two tailed statistical tests and values less than 0.05 were considered statistically significant. Each polymorphism was tested for Hardy-Weinberg equilibrium in the case and control population using *χ*^2^ test. The SNPStat online software was used to estimate pairwise linkage disequilibrium (LD) and haplotype frequency (https://www.snp-stats.net/snpstats).

## Results

Our results showed that of 141 endometriosis patients, 100 (71.0%) and 25 (17.7%) had stage I–II and III–IV of the disease, respectively; on the other hand, in 16 (11.3%) patients, the severity of the disease was not determined. The mean age of healthy and endometriosis group was 29±5 (range 19–44) and 31±6 (19–50) years, respectively that showed significant differences (p=0.03). The Body Mass Index (BMI) comparison between two groups shows significant differences (p= 0.01). The differences of age and BMI between groups could affect the other results and therefore the genotype analysis was modified with regression logistic analysis based on age and BMI.

The distributions of genotype using *χ*^2^ shown in both case and control groups for the *NAT2* C481T, G590A, A803G and G857A polymorphisms were in Hardy-Weinberg equilibrium (p>0.05).

The genotype distributions and allele frequencies of the *NAT2* C481T, G590A, A803G and G857A polymorphisms have been shown in table 1. Our results revealed that there was a significant difference in the genotype distributions and allele frequencies of *NAT2* G590A polymorphism between the case and control groups. The *NAT2* 590 GA genotype was significantly lower (p=0.001; OR=0.42, 95% CI: 0.25–0.71) in the patients (38.3%) than the control group (55.1%). The *NAT2* 590A allele was significantly lower (p=0.033; OR=0.69, 95% CI: 0.49–0.79) in the patients (31.2%) compared with the controls (39.6%). This finding suggested that the *NAT2* G590A polymorphism may be associated with susceptibility to endometriosis and the 590A allele may have a protective role in the development of endometriosis in Iranian women.

**Table 1.** Allele and genotype frequencies of *NAT2* C481T, A803G, G857A and G590A polymorphisms in endometriosis patients and controls

**SNPs ID**	**Genotype/Allele**	**Cases (n=141)**	**Controls (n=158)**	**p-value**	**OR (95% CI)**
**rs1799929 (C481T)**					
	CC	37(26.2%)	38 (24.0%)	**Reference Genotype**					
	CT	77 (54.6%)	88 (55.7%)	0.794	1.08 (0.59–1.97)
	TT	27(19.2%)	32 (20.3%)	0.289	0.67 (0.32–1.40)
	C	151 (53.6%)	164 (51.9%)	**Reference Allele**					
	T	131 (46.4%)	152 (48.1%)	0.687	0.94 (0.68–1.3)
**rs1799930 (G590A)**					
	GG	70 (49.6%)	52 (32.9%)	**Reference Genotype**					
	GA	54 (38.3%)	87 (55.1%)	**0.001**	0.42 (0.25–0.71)
	AA	17 (12.1%)	19 (12%)	0.159	0.67 (0.33–1.40)
	G	194 (68.8%)	191 (60.4%)	**Reference Allele**					
	A	88 (31.2%)	125 (39.6%)	**0.033**	0.69 (0.49–0.97)
**rs1208 (A803G)**					
	GG	58 (41.1%)	65 (41.1%)	**Reference Genotype**					
	GA	58 (41.2%)	73 (46.2%)	0.659	0.84 (0.39–1.81)
	AA	25 (17.7%)	20 (12.7%)	0.638	0.64 (0.30–1.37)
	G	108 (38.3%)	113 (35.8%)	**Reference Allele**					
	A	174 (61.7%)	113 (64.97%)	0.520	
**rs1799931 (G857A)**					
	GG	73 (51.8%)	74 (46.8%)	**Reference Genotype**					
	GA	63 (44.7%)	72 (45.6%)	0.481	0.84 (0.52–1.37)
	AA	5 (3.5%)	12 (7.6%)	0.086	0.37 (0.12–1.85)
	G	209 (74.1%)	220 (69.6%)	**Reference Allele**					
	A	73 (25.9%)	96 (30.4%)	0.223	0.8 (0.56–1.10)

Adjusted by age and BMI

In contrast, no significant difference in the genotype and allele frequencies of the *NAT2* C481T, A803G and G857A polymorphisms was found between the case and control groups.

The frequency of *NAT2* acetylator phenotypes are shown in table 2. Slow phenotype was prevalent in the control group. Statistical analysis showed that the frequency of rapid acetylator phenotype was significantly higher in the endometriosis (34.8%) than the control (27.8%) group (p=0.025; OR=2.19, 95% CI: 1.10–4.36). Analysis of haplotypes showed that among different combinations of the four *NAT2* SNPs (Table 3), only haplotype (481C, 803A, 590A, 587A) was significantly different between the case and control women (p=0.029; OR=3.11, 95% CI: 1.13–8.52).

**Table 2.** The *NAT2* acetylator phenotypes in cases and controls

**Phenotype**	**Cases (n=141)**	**Controls (n=158)**	**p-value**	**OR (95% CI)**
**Slow**	37 (26.2%)	62 (39.3%)	**Reference Phenotype**	
**Intermediate**	55 (39.0%)	52 (32.9%)	0.136	1.65 (0.86–3.21)
**Rapid**	49 (34.8%)	44 (27.8%)	0.025	2.19 (1.10–4.36)

**Table 3.** Haplotypes frequency and association with endometriosis (n=299)

**C481T**	**A803G**	**G590A**	**G857A**	**Frequency**	**OR (95% CI)**	**p-value**
**T**	G	G	G	0.179	1.00	
**C**	A	A	G	0.149	1.03 (0.49 – 2.15)	0.94
**C**	A	G	G	0.139	1.44 (0.70–2.96)	0.33
**T**	A	A	G	0.079	1.07 (0.47–2.48)	0.85
**T**	A	G	A	0.071	1.69 (0.65 – 4.37)	0.28
**C**	**A**	**A**	**A**	**0.071**	**3.11 (1.13 – 8.52)**	**0.029**
**C**	G	G	G	0.068	0.42 (0.13 – 1.37)	0.15
**T**	A	G	G	0.066	1.21 (0.45 – 3.25)	0.70
**C**	A	G	A	0.055	0.20 (0.03 – 1.22)	0.081
**T**	G	G	A	0.051	1.09 (0.34 – 3.54)	0.88
**T**	G	A	G	0.019	3.12 (0.25 – 38.97)	0.38
**C**	G	A	G	0.018	0.85 (0.13 – 5.67)	0.87

## Discussion

C481T, G590A, A803G and G857A polymorphisms in exon 2 of *NAT2* with the risk of endometriosis were investigated in an Iranian population. Our results revealed that there was significant difference in the genotype distributions and allele frequencies of *NAT2* G590A polymorphism between the women with endometriosis and control groups. The *NAT2* 590 GA genotype and *NAT2* 590A allele were significantly lower in the patients than the controls (p=0.001 and p=0.033, respectively). This finding suggested that the polymorphism may be associated with susceptibility to endometriosis and *NAT2* 590A allele may have a protective role in the progression of endometriosis in Iranian women. On the other hand, the frequency of rapid acetylator phenotype was two-fold higher in the endometriosis than the normal controls.

The global Minor Allele Frequency (MAF) in the NCBI dbSNP database of the *NAT2* C481T, G590A, A803G and G857A polymorphisms were T=0.27, A= 0.27, G=0.32 and A=0.08, respectively. According to these frequencies, G857A is a relatively rare polymorphism.

The results of a few studies that evaluated the association of *NAT2* polymorphisms and acetylator phenotypes with the endometriosis are controversial 19–23. There may be several explanations for the observed discrepancies. For example, in some studies only endometriosis patients with stage I–II or III–IV have been included. On the other hand, in some studies the acetylator phenotype for *NAT2* enzyme was classified as slow and fast, while in the others, a three-category classification as slow, intermediate and fast has been used.

Baranova *et al* have reported that a higher proportion of women with stage I–II endometriosis were slow acetylators compared with controls, who were women undergoing termination of pregnancy with no evidence of endometriosis on physical or ultrasound examination (69 *vs*. 39%) 21. There was no statistically significant difference in acetylator status between women with stage III–IV endometriosis and the controls 20. Bischoff *et al* only investigated women with stage III–IV endometriosis and reported that 16/29 (55%) had the slow-acetylator phenotype 20.

Nakago *et al* investigated the relationship between endometriosis and *NAT2* polymorphisms in a UK population 18. Homozygotes for the *NAT2* *4 wild type allele are fast *NAT2* acetylators, while heterozygotes with one wild-type allele and a variant *NAT2* *5, *6 or *7 allele have reduced enzyme activity, and individuals with two variant alleles are slow acetylators. The *NAT2* *4/*6 genotype was significantly more common among endometriosis patients (35.2%) than the controls (8.1%) or unaffected women (4.2%). Fast acetylators phenotype was significantly prevalent in endometriosis group (57.4%) than the controls (32.3%) or unaffected women (33.3%). Their results suggest that altered *NAT2* enzyme activity may be a predisposition factor in endometriosis 18.

Babu *et al* investigated the *NAT2* polymorphisms in 252 unrelated women with endometriosis and 264 controls of South Indian women 22. They found no differences between the frequencies of fast and slow acetylators in cases (34.9 and 65.1%) and controls (33.3 and 66.7%). Deguchi *et al* investigated the association between endometriosis and polymorphisms in the *NAT1* and *NAT2* in a Japanese population 23. Their results showed that the distribution of *NAT1* and *NAT2* allele and genotype frequencies were not significantly different between Japanese cases and controls.

## Conclusion

Investigation of *NAT2* polymorphisms (C481T, A803-G, G857A and G590A) showed that there may be an association between G590A polymorphism and risk for development of endometriosis in Iranian population, and the 590A allele may have a protective role in development of endometriosis. The *NAT2* 481C, 803A, 590A, 587A haplotype was associated with a higher risk of endometriosis.

## References

[1] Bellelis P Podgaec S Abrão MS Environmental factors and endometriosis. Rev Assoc Med Bras 2011;57(4): 448–452. 21876930 10.1590/s0104-42302011000400022

[2] Giudice LC Kao LC Endometriosis. Lancet 2004;364 (9447):1789–1799. 15541453 10.1016/S0140-6736(04)17403-5

[3] Viganò P Parazzini F Somigliana E Vercellini P Endometriosis: epidemiology and aetiological factors. Best Pract Res Clin Obstet Gynaecol 2004;18(2):177–200. 15157637 10.1016/j.bpobgyn.2004.01.007

[4] Nnoaham KE Hummelshoj L Kennedy SH Jenkinson C Zondervan KT World Endometriosis Research Foundation Women’s Health Symptom Survey Consortium Developing symptom-based predictive models of endometriosis as a clinical screening tool: results from a multicenter study. Fertil Steril 2012;98(3):692–701.e5. 22657249 10.1016/j.fertnstert.2012.04.022 PMC3679490

[5] Kennedy S Is there a genetic basis to endometriosis? Semin Reprod Med 1997;15(3):309–318. 10.1055/s-2008-1068761 9383840

[6] Mathur SP Autoimmunity in endometriosis: relevance to infertility. Am J Reprod Immunol 2000;44(2):89–95. 10994636 10.1111/j.8755-8920.2000.440204.x

[7] Berkkanoglu M Arici A Immunology and endometriosis. Am J Reprod Immunol 2003;50(1):48–59. 14506928 10.1034/j.1600-0897.2003.00042.x

[8] Zondervan KT Cardon LR Kennedy SH The genetic basis of endometriosis. Curr Opin Obstet Gynecol 2001; 13(3):309–314. 11396656 10.1097/00001703-200106000-00011

[9] Bischoff F Simpson JL Genetic basis of endometriosis. Ann N Y Acad Sci 2004;1034:284–299. 15731320 10.1196/annals.1335.030

[10] Simpson JL Bischoff FZ Kamat A Buster JE Carson SA Genetics of endometriosis. Obstet Gynecol Clin North Am 2003;30(1):21–40, vii. 12699256 10.1016/s0889-8545(02)00051-7

[11] Hickman D Risch A Buckle V Spurr N Jeremiah S McCarthy A Chromosomal localization of human genes for arylamine N-acetyltransferase. Biochem J 1994;297(Pt 3):441–445. 8110178 10.1042/bj2970441 PMC1137852

[12] Sim E Walters K Boukouvala S Arylamine N-acetyl-transferases: from structure to function. Drug Metab Rev 2008;40(3):479–510. 18642144 10.1080/03602530802186603

[13] Hein DW Boukouvala S Grant DM Minchin RF Sim E Changes in consensus arylamine N-acetyltransferase gene nomenclature. Pharmacogenet Genomics 2008;18 (4):367–368. 18334921 10.1097/FPC.0b013e3282f60db0 PMC2386960

[14] Risch A Wallace DM Bathers S Sim E Slow N-acetylation genotype is a susceptibility factor in occupational and smoking related bladder cancer. Hum Mol Genet 1995;4(2):231–236. 7757072 10.1093/hmg/4.2.231

[15] Cartwright RA Glashan RW Rogers HJ Ahmad RA Barham-Hall D Higgins E Role of N-acetyl-transferase phenotypes in bladder carcinogenesis: a pharmacogenetic epidemiological approach to bladder cancer. Lancet 1982;2(8303):842–845. 6126711 10.1016/s0140-6736(82)90810-8

[16] Ilett KF David BM Detchon P Castleden WM Kwa R Acetylation phenotype in colorectal carcinoma. Cancer Res 1987;47(5):1466–1469. 3815349

[17] Smelt VA Mardon HJ Sim E Placental expression of arylamine N-acetyltransferases: evidence for linkage disequilibrium between NAT1*10 and NAT2*4 alleles of the two human arylamine N-acetyltransferase loci NAT1 and NAT2. Pharmacol Toxicol 1998;83(4):149–157. 9820875 10.1111/j.1600-0773.1998.tb01461.x

[18] Kuznetsov IB McDuffie M Moslehi R A web-server for inferring the human N-acetyltransferase-2 (NAT2) enzymatic phenotype from NAT2 genotype. Bioinformatics 2009;25(9):1185–1186. 19261719 10.1093/bioinformatics/btp121 PMC2672629

[19] Nakago S Hadfield RM Zondervan KT Mardon H Manek S Weeks DE Association between endometriosis and N-acetyl transferase 2 polymorphisms in a UK population. Mol Hum Reprod 2001;7(11):1079–1083. 11675475 10.1093/molehr/7.11.1079

[20] Bischoff FZ Marquez-Do D Kosugi Y Mitchell-Leef D Malinak LR Simpson JL Association of N-acetyltransferase 2 (NAT2) genetic polymorphism resulting in decreased capacity to detoxify aromatic amines in women with endometriosis. J Soc Gynecol Investig 1998;11001(5):111A.

[21] Baranova H Canis M Ivaschenko T Albuisson E Bothorishvilli R Baranov V Possible involvement of arylamine N-acetyltransferase 2, glutathione S-transferases M1 and T1 genes in the development of endometriosis. Hum Reprod 1999;5(7):636–641. 10.1093/molehr/5.7.636 10381818

[22] Babu KA Rao KL Reddy NG Kanakavalli MK Zondervan KT Deenadayal M N-acetyl transferase 2 polymorphism and advanced stages of endometriosis in South Indian women. Reprod Biomed Online 2004;9 (5):533–540. 15588473 10.1016/s1472-6483(10)61638-0

[23] Deguchi M Yoshida S Kennedy S Ohara N Motoyama S Maruo T Lack of association between endometriosis and N-acetyl transferase 1 (NAT1) and 2 (NAT2) polymorphisms in a Japanese population. J Soc Gynecol Investig 2005;12(3):208–213. 10.1016/j.jsgi.2005.01.008 15784508

